# 1925. Prevalence of SARS-CoV-2 Infection in Immunocompromised Patients Following Receipt of Tixagevimab/Cilgavimab

**DOI:** 10.1093/ofid/ofac492.1552

**Published:** 2022-12-15

**Authors:** Sarah Lim, Keeley Morris, Lydia J Fess, Kathy Como-Sabetti, Ruth Lynfield

**Affiliations:** Minnesota Department of Health, St. Paul, Minnesota; Minnesota Department of Health, St. Paul, Minnesota; Minnesota Department of Health, St. Paul, Minnesota; Minnesota Department of Health, St. Paul, Minnesota; Minnesota Department of Health, St. Paul, Minnesota

## Abstract

**Background:**

Tixagevimab/cilgavimab (TC) was approved by the FDA in December 2021 for use as pre-exposure prophylaxis in patients with moderate to severe immune compromise. On February 24, FDA recommended a second dose for patients who received the original dosing because of decreased activity against Omicron subvariants. We were interested in reviewing TC experience in Minnesota.

**Methods:**

Minnesota Department of Health established a voluntary TC patient registry in December 2021, including date of treatment, COVID-19 vaccination status and immunocompromising conditions. Patients were matched to state COVID-19 case data from December 1, 2021 to April 22, 2022, to examine occurrence of SARS-CoV-2 infection (a positive test by PCR or antigen) following receipt of TC.

**Results:**

Data were available for 289 patients, representing 5-10% of all patients treated with TC in Minnesota. 53% were male with a median age of 62 (IQR 48-70). 13 patients (4.5%) had not received COVID-19 vaccine at the time of initial TC dose. 128 patients (44%) received 2 doses of TC. Immunocompromising conditions included: hematological malignancy (114, 39.4%), treatment with immunosuppressant medications (113, 39.1%), solid organ transplant (45, 15.6%), and stem cell transplant (13, 4.5%). 5 patients (1.7%) had a positive SARS-CoV-2 test (4 PCR, 1 antigen) following receipt of TC (Table 1); patients tested positive on days 7, 11, 13, 17 and 48/70. Three patients were on rituximab and 2 had hematological malignancy. All 5 had received 3 doses of COVID-19 vaccine prior to receipt of TC. Variant information was available for 2 patients: BA.1 and BA.1.1. 1 patient required hospitalization for COVID-19 and died 39 days after the positive test but had 3 subsequent negative tests before discharge; death was attributed to underlying malignancy.

**Table 1:**
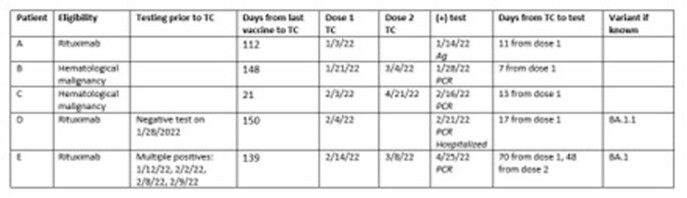
Characteristics of patients with positive SARS-CoV-2 tests following treatment with tixagevimab/cilgavimab.

**Conclusion:**

In a convenience sample of 289 patients who received TC, 5 patients had COVID-19, with 3 occurring within the SARS CoV-2 incubation period following receipt of TC. One of the other patients was positive after receiving 2 doses of TC. Following effectiveness of TC will be useful as SARS CoV-2 continues to evolve.

**Disclosures:**

**All Authors**: No reported disclosures.

